# Effects of a psychological intervention programme on mental stress, coping style and immune function in percutaneous coronary intervention patients

**DOI:** 10.1371/journal.pone.0187745

**Published:** 2018-01-22

**Authors:** Xiaoying Shen, Xuemei Zhu, Yanni Wu, Yuqiu Zhou, Li Yang, Yini Wang, Qiulan Zheng, Yinghui Liu, Shen Cong, Ningning Xiao, Qiuli Zhao

**Affiliations:** 1 College of Nursing, Harbin Medical University, the 2nd Affiliated Hospital of Harbin Medical University, Harbin, China; 2 Nursing Department, the 2nd Affiliated Hospital of Harbin Medical University, Harbin, China; 3 College of Nursing, Daqing Campus of Harbin Medical University, Daqing, China; Public Library of Science, UNITED KINGDOM

## Abstract

**Purpose:**

This study aimed to assess the effects of a psychological intervention programme on the mental stress, coping style and cortisol and IL-2 levels of patients undergoing percutaneous coronary intervention (PCI).

**Methods:**

A total of sixty cardiovascular patients scheduled for PCI with clear anxiety and depression screened by the Hospital Anxiety and Depression Scale were randomly divided into an experimental (n = 30) and control (n = 30) group. The participants in the experimental group received cognitive therapy, relaxation therapy and emotional support. Self-reported questionnaires, including the Self-Report Symptom Checklist (SCL-90) and the Medical Coping Mode Questionnaire (MCMQ), and levels of IL-2 and cortisol were collected at baseline and the day before discharge.

**Results:**

Compared with the controls, patients in the intervention group had a better mental state and coping style (confrontation), higher levels of IL-2 and lower levels of cortisol (*all P<*0.05).

**Conclusions:**

The psychological intervention programme effectively improved mental state, reduced negative coping styles, increased levels of IL-2, and decreased cortisol levels in patients undergoing PCI. This programme may be an effective preoperative nursing intervention for PCI patients.

**Trial registration:**

Chinese Clinical Trail Registry ChiCTR-IOR-16007864

## Introduction

Coronary artery disease (CAD), which is also known as coronary heart disease (CHD), ischaemic heart disease (IHD), and atherosclerotic heart disease, is the most common type of heart disease, [1]. This condition is caused by a build-up of plaque along the inner walls of the arteries of the heart, which reduces blood flow to the heart by narrowing or closing the arteries, resulting in various clinical symptoms, such as angina, arrhythmia, myocardial infarction, congestive heart failure, and even sudden cardiac death.

CAD is the leading cause of death globally, with an incidence of 1.4%, and accounts for approximately 12 million deaths each year. In China, along with the improvement in living standards and changing lifestyles, the prevalence and mortality rates of CAD have increased [2, 3]. According to the Ministry of Health, 750 thousand new cases of CAD occur in China each year [4]. Thus, cardiovascular disease has become an important cause of death in China.

Percutaneous coronary interventions (PCIs), such as percutaneous transluminal coronary angioplasty (PTCA) and intracoronary stenting, constitute a major achievement in interventional cardiology, as they provide immediate relief of symptoms and improve the functional capacity of CAD patients. Many studies have reported the advantages of PCI, which include minimal trauma, low morbidity and mortality, and short periods of hospitalization and convalescence [5,6]. In China, the number of patients undergoing PCI has been increasing at a rate of 30%-40% per year [7]. PCI has been widely accepted as a safe and effective diagnostic and treatment technique. However, it may increase the risk of preoperative stress [8–12], as it is an invasive technique, and patients might be unfamiliar with this treatment. Thus, several reports have investigated the psychological factors of PCI patients.

Many studies have shown that there is a widespread stress response in patients undergoing coronary intervention, predominantly anxiety and other negative emotions.

Negative emotions can interfere with patient understanding of a disease, weaken coping and adaptive strategies, and consequently affect quality of life and rehabilitation. Anxiety, stress, and insomnia significantly affected CAD treatment and led to an increase in the infarction area and even arrhythmia [13]. If a timely intervention is not provided to CAD patients, the mortality rate increases 3-fold, while for patients with hypertension and panic attacks, the probability of sudden death increases 4 to 6 times [14].

Along with social and economic developments and improvements in medical technology, people's quality of life has improved, life expectancy has been extended, the ageing of the society has accelerated, and the incidence of cardiovascular disease has increased. PCI is equivalent to surgical treatments as a proven and effective treatment. However, many patients often suffer from dysfunction of the nervous, endocrine, and circulatory systems and from abnormal psychological conditions, which can lead to postoperative complications and can affect patient prognosis.

To the best of our knowledge, current studies on psychological interventions in patients undergoing PCI have focused on cognitive behaviour. To date, no previous studies have focused on a comprehensive psychological intervention for patients undergoing PCI and have assessed the efficacy of the intervention on biological indexes (e.g., level of IL-2 and cortisol). Therefore, we intended to develop a comprehensive psychological intervention, which incorporated cognitive intervention, relaxation training, behavioural intervention, psychological support, emotional support, and social support, that was accessible to patients undergoing PCI. The present paper presents the effects of this intervention on mental stress, coping style and immune function among patients undergoing PCI. We hypothesized that this intervention would lead to an improvement in adaption, coping, and endocrine hormone levels and a reduction in symptoms of psychological stress.

## Materials and methods

### Ethics statements

All subjects provided written informed consent to participate in the research (S1 and S2 Files). The study was approved by the Ethics Committee of the College of Nursing of Harbin Medical University (S3–S6 Files).

### Study design

A randomized controlled trial was used to assess the effects of a comprehensive psychological intervention on mental stress, coping style and immune function in patients undergoing PCI. This study was registered at http://www.chictr.org.cn/showprojen.aspx?proj=13189. No related trials have been registered, and further trials will be prospectively registered in the future.

### Participants

The sample size for this study was calculated using the following formula: N = $\frac{\left(r+1\right)}{r}\frac{({Z_{\alpha /2}+Z_{1-\beta })}^{2}\sigma^{2}}{{}^{d^{2}}}$. r is the ratio of the sample size between the experimental group and the control group, in the study, r was 1. The significance level (α) was set at 0.05, the Z_α/2_ is 1.96 (two tailed); the statistical power (1-β) was set at 0.9, the Z_β_ is 1.282. According to the relevant literature of anxiety, which was one of the main outcomes in this study, the standard deviation (σ) was set at 2.2; and it was assumed that a mean difference of 2 points (d = 2) can be obtained by the intervention [15]. Then the sample size of each group was calculated to be 25. Taking into account the 20% loss, finally, the sample size was expanded to 30 in each group.

**Fig 1:**
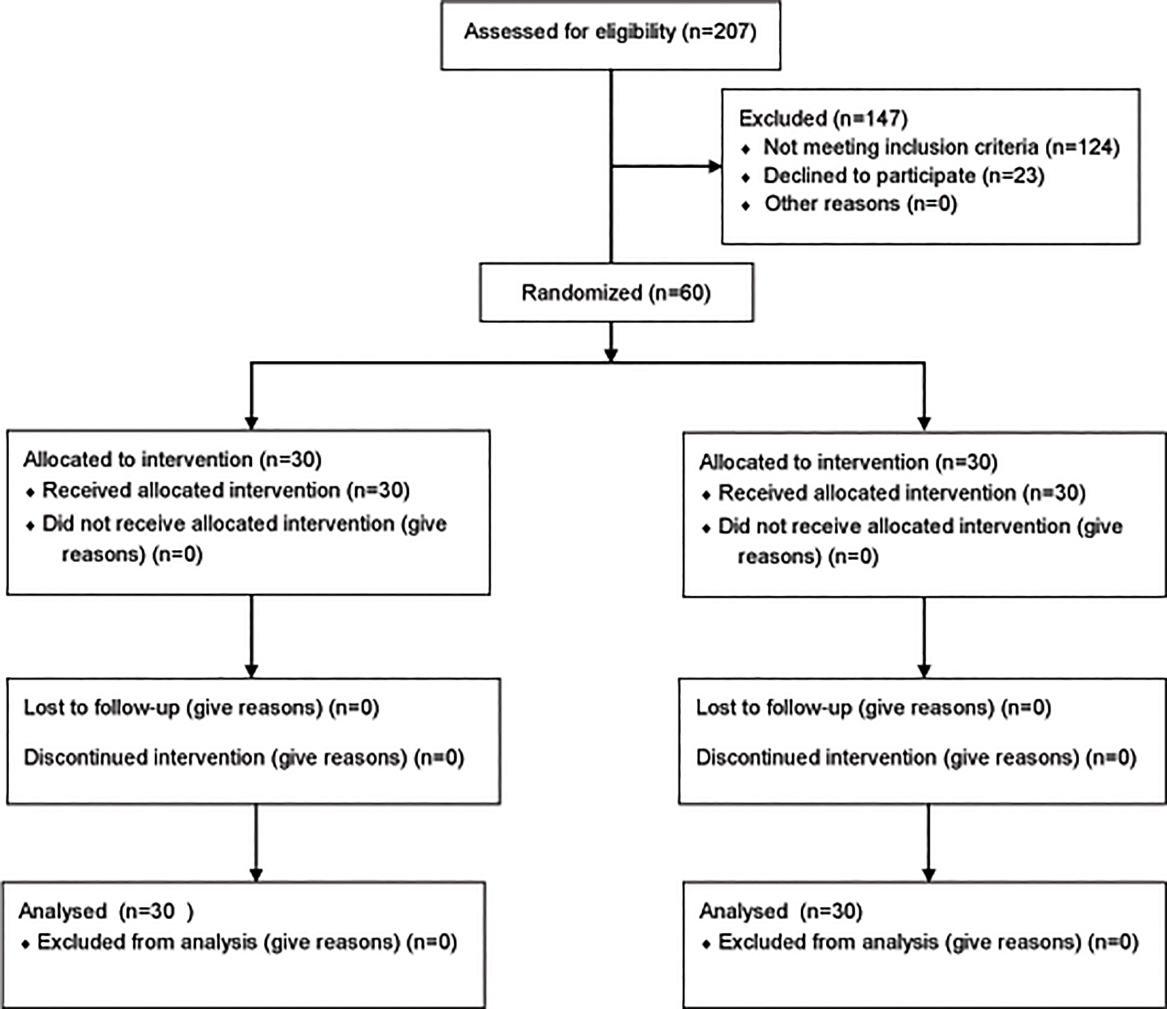
CONSORT 2010 flow diagram.

Then, a cohort of 60 patients was recruited and screened from the four cardiovascular inpatient departments of a teaching hospital located in the northeast PRC, from August 2013 to May 2014. The inclusion criteria included individuals who 1) were diagnosed with angina pectoris or myocardial infarction, had an ECG showing ST elevation, had stable vital signs and were scheduled for PCI for the first time; 2) were 18–75 years of age; 3) were conscious and able to communicate in Chinese; and 4) scored greater than 8 on the Hospital Anxiety and Depression Scale (HADS, the Chinese version of HADS has been validated [16]) on the day of admission. Patients who suffered from other serious circulatory system diseases or had serious complications, had other serious somatic or mental illnesses, were cognitively impaired, or had primary or secondary hypercortisolism and cortisol thrombocytopenia were excluded (Fig 1, S7 File).

### Procedure

A two-stage procedure was used to select and randomize participants. Sixty consecutive patients meeting the inclusion criteria who were routinely scheduled for PCI and consented to participate in the study were chosen. Next, we randomly assigned the participants at a ratio of 1:1 to either the experimental or a control group by random sequence number generation. The random number sequence was generated by the project leader, Xiaoying Shen, using a computer. All subjects were assigned to a different group by a random number; those with an odd number entered the experimental group, and those with an even number were in the control group. All participants were given a small gift (RMB 50 Yuan or shopping card of equivalent value) after participation.

#### Standard care

Both the control and the experimental group received the same standard nursing care, consisting of preoperative preparation, care of the drainage tube and incision site, and health education concerning diet and activities. Additionally, this treatment included an explanation of the cause of disease, the purpose and process of the operation, postoperative cautions based on age, and education about the disease.

#### The psychological intervention programme

In addition to the usual care described above, patients allocated to the intervention group were offered a psychological intervention programme. The intervention had three specific components: 1) cognitive therapy; 2) relaxation therapy; and 3) emotional support. Table 1 presents the outline of the intervention. The protocol was present in S8 and S9 Files.

**Table 1:** Contents of intervention sessions.

Session	Contents	Temporal arrangement
**1. Cognitive** **therapy**	(a) Organization of a meeting for postoperative and preoperative patients to encourage the preoperative patients to accept the treatment and operation positively and optimistically. (b) Researcher played a videotape about the operation process and the postoperative precautions by patient’s bedside.	The day of the decision regarding the operation (30–45 min) The day before the operation (30 mins)
**2. Relaxation** **therapy**	Consisted of progressive muscle relaxation, meditation, thought guidance, deep breathing, and massage by a specific counselling expert. Note: during the training period, the participants needed to relax the whole body and concentrate.	Each morning and afternoon of hospitalization voluntarily, 15–30 min each time
**3. Emotional support**	Created a relaxing and warm environment for patients and families and gave them moral encouragement and appropriate daily care. Encouraged patients and families to communicate with each other. In terms of social support, invited post-PCI patients to impart their experiences to patients.	Daily communication with patients and family

#### Outcome measures

Demographics, medical history data and clinical data were collected by researcher-designed questionnaires. There were four demographic questions concerning age, gender, marriage, and education and one medical history question, whether they had an endovascular stent. Five laboratory values were recorded from the medical record (haemoglobin, total cholesterol, others). The participants completed the following self-report assessments (SCL-90 and MCMQ) two times over the course of the study: prior to the start of the programme (pre-test), i.e., on the day of admission, and after completion of the programme (post-test), i.e., the day before discharge. Immune outcomes (IL-2 and cortisol levels) were determined the day after admission and the day before discharge. All questionnaires were distributed by the same person one by one and face to face. Where there is doubt about the questionnaires, an unbiased interpretation would be given by the investigator.

Mental stress was measured using the SCL-90 assessment. The SCL-90 was established by L.R. Derogatis in 1975. Then it has been widely used in psychiatric and psychological counselling departments and in the general hospital, which has also investigated the mental health problems of different groups from different perspectives. The Chinese version of SCL-90 used in this study was translated by Zhengyu Wang [17,18]. It is composed of 90 self-reported items, 9 factors on symptoms and 1 factor that assesses additional items. This scale contains various items on feeling, thinking, awareness, behaviour, life habits, interpersonal relationships, diet, and sleep, among others. The ten factors are somatization, obsessive-compulsive, interpersonal sensitivity, depression, anxiety, hostility, paranoid ideation, psychoticism, phobic anxiety, and other. The Chinese version has been validated, and the Cronbach’s α coefficient of the dimensions has been found to be 0.69–0.86, and the test-retest reliability was 0.73–0.91. The correlation coefficient between each dimensions and the scale was 0.79–0.92 and the correlation coefficient between each dimensions was 0.59–0.83 [19].

Coping style was measured using the MCMQ assessment. The MCMQ was provided by Feifel H, it contains 20 items covering three dimensions, namely confrontation, avoidance, and suppression, which reflect the basic reactions of people at risk [20]. The Chinese version was revised, analysed and tested by Qianjin Jiang and Xiaohong Shen. In the Chinese version we used, the Cronbach’s α coefficient of the three dimensions has been found to be 0.69, 0.60 and 0.76, respectively, and the test-retest reliability was 0.64, 0.85, and 0.67, respectively. The correlation coefficient between confrontation and avoidance is 0.14, between confrontation and suppression is 0.15, and between avoidance and suppression 0.03. These values indicate that the classification of coping styles is appropriate [21].

IL-2 and cortisol were measured in the blood at two time points as described previously. The IL-2 and cortisol samples were collected at 6 AM, when the patients had an empty stomach, to avoid physiological for analysisy mission)ping stying stype. ts needed to relax the whole body muscle and concentration. fluctuations. Blood samples were sent to the endocrine laboratory for analysis. For cortisol, an iodine (125I) glycol (Cor) RIA kit was provided by the Beijing Kemeidongya Biological Technology Co., Ltd.

#### Data analysis

Data were analysed using the PASW Statistic 17.0 Programme (SPSS Inc., Chicago, IL, USA) by another researcher who was blinded to the allocation. The baseline characteristics of the subjects were analysed using raw numbers and percentages. Homogeneity tests of the baseline characteristics of the intervention and control groups were performed using independent *t*-tests and chi-square analyses. At the pre-test period, no significantly different characteristics were found between the two groups. Therefore, independent sample *t*-tests were used to evaluate the outcome variables to determine the effectiveness of the intervention. We set the power of the study at 0.50. For all analyses, missing data were transformed by mean imputation [22], and P<0.05 was considered significant.

In this study, Cohen’s d for paired samples was used to represent the effect size of control group or intervention group. Generally, the value of Cohen’s d = ±0.20, is typically used to represent a ‘‘small” effect size, ±0.50 a ‘‘medium” effect size, and ±0.80 a ‘‘large” effect size [23]. Effect size represents the difference between a treatment group and the control group. Medium to large effect sizes are generally suggested for behavioral research [24].

## Results

### Homogeneity test of subjects

As shown in Table 2, the demographic and clinical characteristics of the participants was not different significantly between the two groups at the pre-test period (all P>0.05). The mean age of the study participants was 57.6 (SD = 9.9) years, and the majority had a junior high school education level (60.0%). In total, 45.0% of the patients had endovascular stents.

**Table 2:** Demographic and clinical characteristics of the participants (N = 60).

Characteristic		Total (n = 60)	Intervention group (n = 30)	Control group (n = 30)	Pvalue
**Gender**	**Male**	30 (50.0%)	16 (53.3%)	14 (46.7%)	0.80
	**Female**	30 (50.0%)	14 (46.7%)	16 (53.3%)	
**Age**		57.6±9.92	58.6±10.7	56.5±9.2	0.41
**Education level**	**Junior high school**	36 (60.0%)	18 (60.0%)	18 (60.0%)	0.47
	**Senior high school**	13 (21.7%)	8 (26.7%)	5 (16.7%)	
	**College**	11 (18.3%)	4 (13.3%)	7 (23.3%)	
**Haemoglobin (mmol/L)**		8.17±1.25	8.27±1.37	8.06±1.19	0.53
**Total cholesterol (mmol/L)**		5.24±0.54	5.23±0.57	5.26±0.52	0.86
**Triglyceride (mmol/L)**		1.97±0.33	2.04±0.34	1.91±0.30	0.14
**High-density lipoprotein cholesterol (mmol/L)**		1.64±0.49	1.59±0.51	1.68±0.47	0.49
**Low-density lipoprotein cholesterol (mmol/L)**		2.82±0.43	2.86±0.44	2.79±0.42	0.57
**Endovascular Stents**	**Yes**	27 (45.0%)	15	12	0.44
	**No**	33 (55.9%)	15	18	

At the pre-test period, the mean score of mental stress measured by the SCL-90 was 192.55 (SD = 38.06), the total coping style score measured by the MCMQ was 48.82 (SD = 3.68), the mean IL-2 level was 30.54 ng/ml (SD = 8.80) and the mean cortisol was 443.88 ng/ml (SD = 71.49) (Table 3).

**Table 3:** Comparison of SCL-90, MCMQ, IL-2 and cortisol between groups before the intervention.

Variable	Total (n = 60)	Experimental (n = 30)	Control (n = 30)	P value
	$\bar{x}$ ±SD	$\bar{x}$ ±SD	$\bar{x}$±SD	
**SCL90**	192.55±38.06	193.87±39.07	191.23±37.65	0.79
**Somatization**	31.40±8.41	31.60±8.52	31.20±8.45	0.86
**Obsessive-compulsive**	23.45±6.02	22.50±5.46	24.40±6.47	0.22
**Interpersonalsensitivity**	14.77±4.93	15.73±5.25	13.80±4.46	0.13
**Depression**	28.15±8.80	28.33±9.33	27.97±8.39	0.87
**Anxiety**	25.73±6.94	25.43±6.42	26.03±7.51	0.74
**Hostility**	10.85±3.74	11.57±3.89	10.13±3.49	0.14
**Fear**	14.43±5.89	15.87±6.60	13.00±4.77	0.06
**Paranoid ideation**	10.13±3.15	9.37±2.77	10.90±3.36	0.06
**Psychoticism**	17.85±4.44	17.43±4.55	18.27±4.36	0.47
**Other**	16.13±5.17	16.03±4.86	16.23±5.55	0.88
**MCMQ**	48.82±3.68	49.50±4.02	48.13±3.22	0.15
**Confrontation**	19.97±2.16	20.07±2.46	19.87±1.83	0.72
**Avoidance**	15.42±2.29	15.80±2.58	15.03±1.92	0.20
**Suppression**	13.25±1.99	13.27±2.27	13.23±1.72	0.95
**IL-2 (ng/ml)**	30.54±8.80	31.54±9.35	29.54±8.26	0.38
**Cortisol (ng/ml)**	443.88±71.49	453.43±67.48	434.32±75.21	0.31

### The effects of the psychological intervention programme on mental stress, coping style and immune function

Independent sample *t*-tests were used to evaluate the effects of the intervention on mental stress, coping style and immune function (Table 4). At the post-test period, compared to the controls, the participants in the experimental group reported significantly lower scores on the SCL-90 and on all dimensions except for sensitivity of interpersonal relationships and fear, higher scores on the MCMQ and the dimension of confrontation, higher IL-2 levels and lower cortisol levels (P< 0.05). These significant values represented mostly moderate effect sizes, with computed Cohen’s d values ranging from ±0.52 to ±1.56 (Table 5).

**Table 4:** Comparison of SCL-90, MCMQ, IL-2 and cortisol between groups after the intervention.

Variable	Experimental (n = 30)	Control (n = 30)	P value
	$\bar{x}$ ±SD	$\bar{x}$ ±SD	
**SCL90**	132.73±20.60	172.57±38.067	0.00
**Somatization**	19.33±4.84	25.97±8.05	0.00
**Obsessive**	16.37±3.16	22.67±6.25	0.00
**Interpersonal sensitivity**	12.50±1.96	14.0±5.26	0.15
**Depression**	18.20±4.70	25.63±7.66	0.00
**Anxiety**	15.17±3.74	21.97±7.02	0.00
**Hostility**	8.13±1.94	10.60±3.10	0.00
**Fear**	11.00±4.06	12.77±5.62	0.17
**Paranoid ideation**	7.20±0.93	8.47±2.56	0.02
**Psychoticism**	13.67±2.44	16.03±3.88	0.01
**Other**	11.17±3.36	14.47±4.81	0.00
**MCMQ**	51.03±3.39	49.13±3.15	0.03
**Confrontation**	21.70±2.02	20.47±1.81	0.02
**Avoidance**	15.53±2.40	15.17±1.72	0.50
**Suppression**	13.80±1.03	13.50±1.46	0.36
**IL-2 (ng/ml)**	35.22±10.52	30.02±8.34	0.04
**Cortisol (ng/ml)**	302.23±79.49	442.41±67.60	0.00

**Table 5:** Effect size of experimental and control group subjects’ pre- versus post-intervention assessments.

	experimental group (n = 30)			Control group (n = 30)		
	Pre-intervention	post-intervention	Cohen’s d*	Pre-intervention	post-intervention	Cohen’s d*
**SCL90**	193.87±39.07	132.73±20.60	-1.56	191.23±37.65	172.57±38.067	-0.50
**Somatization**	31.60±8.52	19.33±4.84	-1.44	31.20±8.45	25.97±8.05	-0.62
**Obsessive-compulsive**	22.50±5.46	16.37±3.16	-1.12	24.40±6.47	22.67±6.25	-0.27
**Interpersonal sensitivity**	15.73±5.25	12.50±1.96	-0.615	13.80±4.46	14.0±5.26	0.04
**Depression**	28.33±9.33	18.20±4.70	-1.09	27.97±8.39	25.63±7.66	-0.28
**Anxiety**	25.43±6.42	15.17±3.74	-1.60	26.03±7.51	21.97±7.02	-0.54
**Hostility**	11.57±3.89	8.13±1.94	-0.88	10.13±3.49	10.60±3.10	0.13
**Fear**	15.87±6.60	11.00±4.06	-0.73	13.00±4.77	12.77±5.62	-0.05
**Paranoid ideation**	9.37±2.77	7.20±0.93	-0.78	10.90±3.36	8.47±2.56	-0.72
**Psychoticism**	17.43±4.55	13.67±2.44	-0.83	18.27±4.36	16.03±3.88	-0.51
**Other**	16.03±4.86	11.17±3.36	-1	16.23±5.55	14.47±4.81	-0.32
**MCMQ**	49.50±4.02	51.03±3.39	0.38	48.13±3.22	49.13±3.15	0.31
**Confrontation**	20.07±2.46	21.70±2.02	0.66	19.87±1.83	20.47±1.81	0.33
**Avoidance**	15.80±2.58	15.53±2.40	-0.1	15.03±1.92	15.17±1.72	0.07
**Suppression**	13.27±2.27	13.80±1.03	0.23	13.23±1.72	13.50±1.46	0.16
**IL-2**	30.61±8.26	34.89±10.47	0.52	31.63±9.34	29.08±8.34	0.27
**Cortisol**	453.43±67.48	302.23±79.49	2.24	434.32±75.21	442.41±67.60	0.11

*: Cohen's d = (M2—M1)⁄ SD preintervention; M2 means the mean of post-intervention; M1 means the mean of the Pre-intervention.

## Discussion

This study was a randomized controlled trial to assess the effect of a psychological intervention programme on mental stress, coping style and levels of cortisol and IL-2 of patients undergoing PCI. This psychological intervention programme improved mental stress and coping style, reduced cortisol, and increased IL-2 in the patients scheduled for PCI for the first time. Therefore, several elements of the psychological intervention programme, such as emotional support, a videotape by patient’s bedside, or peer education, may be used as an independent or routine nursing intervention for patients undergoing PCI. Other elements of the psychological intervention programme, such as relaxation therapy may be used when external conditions permit.

In this study, we confirmed that the levels of anxiety, depression, and other factors of the psychological intervention group were significantly lower than those of the control group; these were also psychological risk markers that are routinely assessed in cardiac patients [25, 26]. Additionally, as shown in Table 4, the intervention group participants improved their total SCL-90 score in the dimensions of somatization, obsession, psychoticism, paranoid ideation, hostility and other symptoms. These results are consistent with previous studies [27 28]. Psychological interventions can significantly improve the mental health status of patients with CAD. They may act synergistically with clinical treatment and can further improve quality of life and prognosis of CHD. Another study investigated the effects (anxiety, sleep, and blood pressure) of aromatherapy on PCI patients in an intensive care unit (ICU). The results showed that aromatherapy effectively reduced the anxiety levels and increased the sleep quality of PCI patients admitted to the ICU [13]. In contrast to that study, our study was carried out in the general wards. PCI patient are not concentrated in one area, and therefore, aromatherapy was not easy to deploy. Our interventions were more easily implemented in the general ward. Additionally, the immune indexes (cortisol and IL-2) we chose appeared to be more stable than blood pressure.

As shown in Table 4, after the intervention in this study, the two groups experienced no changes in avoidance and suppression. However, the intervention group participants had a better confrontation coping style the day before discharge (P < 0.05), which indicates that a comprehensive psychological intervention may improve participants’ coping styles towards confrontation in the short term. In other words, it was difficult for the participants to meaningfully change their avoidance and suppression coping styles over a short intervention period [14, 29]. Generally, confrontation is an active coping style, avoidance and yielding are negative coping styles, and mature coping styles can relieve anxiety to some extent [30, 31]. This result is consistent with several previous studies [10, 11] but not consistent with all [29, 32]. This may be attributed to our short intervention time and small sample size.

IL-2 and cortisol are hormones regulating the immune response stimulated by the adrenal cortex-zona fasciculate. IL-2 is a cytokine produced by T helper cells. It promotes T cell proliferation, enhances the activity of killer cells, and plays an important role in the immune response of the organism. CAD and its related negative emotions act as stressors that stimulate a non-specific response, which leads to a decrease in immune function through the neuroendocrine-immune axis [33]. Stress states can inhibit the generation of IL-2, confirming the effects of psychological factors on human cellular immune function [34]. Appropriate psychological interventions can help relieve patients’ negative emotions [35]. This study confirms that the psychological intervention reduced levels of IL-2 and improved the body’s immune system.

Cortisol has a significant impact on the metabolism of sugar, fat, and protein and tissue and organ function. Psychological stress can active the hypothalamus-pituitary-adrenal (HPA) axis, leading to endocrine disorders [36]. Cortisol, as the last product of the HPA axis, is an indicator of stress. When people are nervous or experience fear and anxiety, stress hormones such as cortisol may be increased. Excess hormones can generate physical reactions, such as significantly increased heart rate, increased blood pressure, quickened breathing, increased myocardial oxygen demand, and decreased coronary blood flow, which can result in myocardial ischaemia and arrhythmia. Cortisol can decrease blood lymphocytes, reduce the activity of T lymphocytes, mononuclear cells and NK cells, reduce the production of cytokines, and reduce the body's immune function. Several researchers have reported that psychological interventions can enhance immunity, improve quality of life, relieve pain and prolong life. Consistent with many previous studies [37,38], our findings indicated that the cortisol levels in the intervention group significantly decreased after the intervention (P<0.01), which indicates that a comprehensive psychological intervention can improve the levels of endocrine hormones through psychological-neuro-immune function, enhance immune function, and decrease heart workload [39].

The small number of participants limits the conclusions that can be drawn from the study. Participants in this study were recruited from only one hospital, which limits the generalizability of the results to all people undergoing PCI. Additionally, the intervention time in this study was short. Thus, future research is required to refine the intervention protocol, and conducting follow-ups to determine the longevity of the effects of the intervention is necessary. Additionally, the levels of cortisol cannot represent overall immune function. How other physiological indexes were affected is unknown. Thus, more research is necessary to assess the dynamic changes in psychological state, coping styles and immune function.

## Conclusion

These data demonstrate an improved outcome of participants assigned to a comprehensive psychological intervention group. The intervention provided psychological support for PCI patients comprehensively and systematically and helped patients adopt positive coping styles gradually in the stressful PCI surgery environment; furthermore, it increased the patients’ serum IL-2 levels and reduced their cortisol levels, restoring the stability of their immune function. The ultimate impact of the intervention is that it helped patients face their disease in the best physiological and mental state and that it enhanced the treatment effects of PCI.

## Supporting information

S1 File Informed consent–Chinese copy. (DOC)

S2 File Informed consent–English copy. (DOC)

S3 File IRB report–Chinese copy. (DOC)

S4 File IRB report–English copy. (DOC)

S5 File Original IRB report page 1. (JPG)

S6 File Original IRB report page 2. (JPG)

S7 File CONSORT 2010 checklist. (DOC)

S8 File Protocol–Chinese copy. (DOCX)

S9 File Protocol–English copy. (DOCX)
