# Supplementary material for: Effects of a psychological intervention programme on mental stress, coping style and immune function in percutaneous coronary intervention patients
Source: PLoS One. 2018 Jan 22;13(1):e0187745. doi: 10.1371/journal.pone.0187745 (PMC5777641; doi:10.1371/journal.pone.0187745)
Supplement: S1 File — (DOC) [file pone.0187745.s001.doc]

**知情同意书**

**题目：**心脏介入诊疗术患者的心理状态分析及综合心理干预模式的研究

**背景信息**

根据您的心脏病病史，您的医生已经建议您参加这项临床研究，在这之前，您有权知道该临床研究的内容以及可能涉及的风险和利益，这份知情同意书详细地记录了这些内容。您可以自愿选择参加或不参加这项研究。我们将在哈尔滨医科大学附属第二医院实施一项关于心脏介入诊疗术患者的心理状态分析及综合心理干预模式的研究。这项临床研究的开展已经获得哈尔滨医科大学生物医学研究伦理委员会的批准，并将在沈晓颖的指导下实施。这项研究入组病人的总数约60人。

**研究背景及目的**

心脏介入性诊断及治疗包括冠状动脉造影、人工心脏起博器安置术、射频消融、经皮穿刺腔内冠状动脉成形术(PTCA)及支架安置术等，已成为治疗心脏病的重要手段。具有创伤小、安全、成功率高、恢复快等优点，现已成为治疗冠心病的主要方法。虽然心脏介入诊疗术创伤较小，患者术后狭窄的冠状动脉得到扩张，心肌灌注得到改善，但作为一种有创性的治疗方法，会对患者造成心理负担，许多患者表现出高度焦虑和抑郁等负性情绪，这些不良的心理反应会直接影响手术过程，使得患者不能很好地应对手术，影响术后康复，因此给予心脏介入手术患者实施有效心理干预，对减轻患者焦虑、恐惧等不良情绪，提高依从性，降低手术并发症的发生率，改善生活质量有重要意义。

目前针对心脏介入手术患者围手术期的护理干预只谈及到心理护理、健康教育和康复指导的其中一个方面，而对患者术前术后综合心理干预目前研究极少。本研究旨在通过对心脏介入手术患者进行综合心理干预（以床旁观看手术录像及情绪疏导为主），即术前术后给予➀认知疗法；➁放松治疗；➂情感支持等一对一式干预方法，使患者有充分的认知，消除焦虑、恐惧和抑郁等负性情绪，提高了病人的心理应对能力，使其身心于最佳状态接受手术，保证手术的顺利进行, 减少术后不适反应的发生，缩短住院时间，做到二级预防。同时降低因手术应激带来皮质醇及白介素2的生化值，提高患者集体免疫功能。

**研究设计和治疗**

这是一项随机化、开放研究，这就是说您不能自行选择治疗组，您将被随机分入综合心理干预组或者常规护理组，两组的观察时间均为7-10天（即一次住院周期）。

在试验期间，将测量以下指标①临床症状自评量表（SCL-90）评定患者的心理健康状况：SCL-90由90个自我评定项目组成。项目分评分标准：采用五级评分法（0～4级）0分=无： 自觉本周内无此问题或症状；1分=轻度：自觉有此问题或症状，但发生得不频繁，程度较轻，持续时间较短：2分=中度：自觉常有此问题或症状，频度和严重度均大于“轻度”。3分=偏重：自觉多数时间均有此问题或症状， 其频度和严重性对自己的学习或生活已产生一定的影响。4分=严重：自觉绝大部分时间都有此问题或症状，自感频度和强度已十分严重。②医学应对问卷（MCMQ）：该医学应对问卷由Feifel H研制，沈晓红等人修订，该量表包含3个维度：面对、回避、屈服，符合人们面临危险事件的基本反应方式，量表含20个条目，条目评分采用Likert 4级评分，从1分“从不这样”到4分“总是这样”，其中有8条为反向记分，各维度由相应条目分累计所得，得分越高代表病人采用该分量表代表的应对方式的倾向性就越大。3个分量表内部一致性系数（a系数）分别为:面对0.69，回避0.60，屈服0.76；重测信度为:面对0.64，回避0.85，屈服0.67；面对与回避、屈服的相关系数分别为0.14、0.15，回避与屈服的相关系数为0.03，显示3种应对的分类是合适的。③实验材料及血液标本的采集：碘[125I]皮质醇（Cor）放射免疫试剂盒 北京科美东雅生物技术有限公司提供。采血时间定于入院后第 2 天和手术后第4 天，为避免生理波动对结果的影响，标本采集时间固定为清晨 6：00 空腹采血，测量皮质醇和白介素2生化值。

**研究程序**

　 如果您同意参加这项研究，您将接受一系列“筛选评价”确保您符合这项研究的所有要求，也是为了在实施这项研究之前确保您的安全，这项筛选评价包括体医学焦虑与抑郁问卷的测量。如果您的所有筛选评价都符合本研究入选的要求，您将被随机分配至实验干预组或者常规护理组，无论您被分入哪一个治疗组，您都将在治疗过程中接受常规护理。

**风险和不适**

不适： 在试验期间，您的主管护师将密切关注您的情况，如果您发生任何不适或反应，请随时通知您的护理人员。以便得到及时的处理。

**受试者收益**

通过综合心理干预，可以改善患者的紧张、焦虑等不良心理状况，可以提高患者面对的应对方式，使患者以更积极的态度面对疾病，更好的配合手术，降低手术并发症，对临床治疗起到协同作用，从而提高患者生活质量。综合心理干预能够通过心理—神经—免疫的作用改善患者的内分泌激素水平，改善PCI患者的心理应激状态，降低皮质醇水和白介素2的水平，从而有效地增强患者的免疫功能，从而减轻心脏负担。有学者认为心理干预可提高免疫力，改善生活质量，减轻痛苦和延长生命。

**记录的机密性**

您的隐私权受到保护，您的个人资料是保密的，只有本项目的实施者、医学伦理委员会及国家食品药品监督管理局在必要时可以查阅，您的相关资料可用于医学研究，但任何情况下都不会出现您的名字。

**自愿参加**

参加本研究是完全自愿的，您可以拒绝参加和/或撤回同意书，并在任何时候中断参与本研究，这不会因为您的退出受到不公平待遇而影响您的正常治疗。如果护士考虑到为了您的安全中断研究对您最有益，可以随时中止本研究，回到常规治疗，申办方可以永久性中止本研究。

**获得进一步信息**

如果您在这项研究期间有任何问题、或者发生任何副作用，您可以在任何时间进行咨询，您可以通过

与您的医师 联系。

**知情同意**

**受试者声明和签署**

本人已经阅读上述信息，了解了本研究的目的以及参加本研究的潜在风险与收益。本人得到了提问的机会，所有问题都已经得到满意的答复。本人同意由研究团队收集和处理关于我的信息。本人也同意可以由心内科处理这些信息，或者转交给任何一个与心内科合作的单位，如果我决定退出研究，我同意在我退出之前收集的信息可以被使用，本人同意心内科在今后的医学研究中使用我的数据，包括我的健康资料。

参加的试验名称： 心脏介入诊疗术患者的心理状态分析及综合心理干预模式的研究 随机号：

**我自愿参加这项研究并签署这份知情同意书。我已经得到一份该知情同意书的复印件。**

**患者姓名（印刷体） 患者签名 日期（年/月/日）**

**患者联系电话： 患者通讯地址：**

**如果患者由于是文盲或者功能障碍不能在ICF上签字。可以由独立见证人（或家属）签署，而不用患者本人签署。**

**独立见证人姓名（印刷体） 独立见证人（签名）**

**日期（年/月/日） 联系电话**

**本人，以下签署人，已经采用适当和容易理解的语言与这名患者或者合法代表讨论了这项研究，据我所知，我已经完全告知上述受试者本研究的性质和潜在利益与风险，受试者也完全理解了该信息。我已经为这名受试者提供了一份签署了姓名与日期的知情同意书复印件。**

**研究者姓名（印刷体） 研究者签名 日期（年/月/日）**

**联系电话**
