# Supplementary material for: Effects of a psychological intervention programme on mental stress, coping style and immune function in percutaneous coronary intervention patients
Source: PLoS One. 2018 Jan 22;13(1):e0187745. doi: 10.1371/journal.pone.0187745 (PMC5777641; doi:10.1371/journal.pone.0187745)
Supplement: S2 File — (DOC) [file pone.0187745.s002.doc]

**Informed consent**

**Title:** Psychological status analysis, establishment and effect evaluation of a comprehensive psychological intervention for patients with cardiac interventional therapy

**Background information**

Your doctor has advised you to take part in this clinical study, based on your heart disease history. Previously, you have right to understand the content of the research and the risks and the benefits involved, this informed consent recorded these content in detail. You can voluntarily choose to participate or not in this study. We will carry out this research in the Cardiology Department, the Second Affiliated Hospital of Harbin Medical University. This clinical study has been approved by the bio-medical ethics committee of Harbin Medical University research, And will be implemented under the guidance of Xiaoying Shen. The total number of enrolled patients in the study was about 60.

**Research background and purpose**

Cardiac interventional diagnosis and treatment include coronary angiography, artificial cardiac pacemaker placement surgery, radiofrequency ablation, percutaneous transluminal coronary artery forming (PTCA) and stent implantation, it has become an important treatment of heart disease. It has the advantages of small trauma, safety, high success rate and quick recovery and it has become the main treatment of coronary heart disease. Although cardiac interventional diagnosis and treatment is of less trauma, the stenosis of the coronary artery was expanded, myocardial perfusion was improved after operation, but as a kind of invasive treatment, it will cause psychological burden on patients. Many patients showed a high degree of anxiety and depression or other negative emotions. These adverse psychological reactions will directly affect the operation process. It causes the patient to be unable to cope well with the operation, and impacts the postoperative rehabilitation. Therefore effective psychological intervention can reduce patient's anxiety, fear and other negative emotions. It also can improve the compliance, reduce the incidence of surgical complications and improve the quality of life.

At present, study on peri-operative nursing intervention for patients with cardiac interventional surgery, only include one aspect of psychological care, health education and rehabilitation guidance. But study on the comprehensive psychological intervention before and after the operation is very little. The aim of this study was to explore the effectiveness of comprehensive psychological intervention. The comprehensive psychological intervention included preoperative and postoperative cognitive therapy, relaxation therapy and emotional support.

**Research design and treatment**

This is a randomized, open study, which means that you can not choose the treatment group, you will be randomly assigned to the comprehensive psychological intervention group or the conventional care group, the two groups of observation time is 7 days.

During the test, the following indicators will be measured. ①Self-reporting Inventory(SCL-90), to assess the patients’ mental health status.It is composed of 90 self-reported items, 9 factors of symptoms and 1 factor can't be named. This scale contains of various of items, covering feeling, thinking, awareness, behavior, life habits, interpersonal relationship, diet, sleep, etc. The ten factors were: somatization, obsessive-compulsive, interpersonal sensitivity, depression, anxiety, hostility, paranoid ideation, psychoticism, and others. ②Medical Coping Modes Questionnaire(MCMQ), The MCMQ contains 20 items, covering three dimensions, that is, confrontation, avoidance, and suppression, which reflect the basic reaction of people at risk. ③Blood specimen: collection time fixed for 6:00 in the early morning when on an empty stomach, to measure cortisol and IL-2.

**Research procedure**

If you agree to take part in this study, You will receive a series of "screening evaluations" to ensure that you meet all the requirements of the study. It is in order to ensure your safety before the implementation. This screening evaluation included physical examination and measurement of anxiety and depression. If your screening evaluation meet the standard of the study, You will be randomly assigned to either an experimental intervention group or a conventional care group.

**Risk and discomfort**

During the test, your nurse in charge will pay close attention to your situation, If you have any discomfort, please feel free to inform your nursing staff, in order to get deal with timely.

**Potential benefit**

Comprehensive psychological intervention can improve the patient's anxiety depression and other negative emotion, can improve the patients’ coping style. It also can improve the compliance, reduce the incidence of surgical complications and improve the quality of life. Comprehensive psychological intervention can improve the patient's endocrine hormone through psychological - nerve - immune axis. That is to say it can reduce cortisol and increase the interleukin 2. Thus, patients’ immune function can be effectively enhanced so as to reduce the burden of the heart.

**Confidentiality**

Your privacy is protected. Your personal information is confidential. Only the executor, medical ethics committee, and State Food and Drug Administration can access when necessary. Your information can be used for medical research, but remain anonymous in any case.

**Voluntary**

To participate in this study is completely voluntary. You may refuse to participate and / or withdraw the consent form, and you can exit the study at any time. You will not get unfair treatment and affect your health due to your interrupt. If your nurse in charge think interruption of research is most beneficial to you considering your safe. The research can be discontinued at any time, and return to routine treatment. The bidder can discontinued the study permanently.

**Further information**

If you have any questions during the study period, or any side effects. You can consult at any time, you can contact your physician through .

**Informed consent**

**Subject declaration and signature**

I have read the information above, and have understood the purpose and potential risk and benefit of the study. I have been given the opportunity to ask questions, and all the doubt have been answered with satisfaction. I have agreed that the research team can collect and process information about me. I also agree that the information can be processed by the cardiology department, or any unit that is cooperating with the cardiology department. If I decided to quit the study, I would agree that the information collected before my dropping out could be used. I agree that cardiology department can use my data for future medical research, including my health data.

Name of the study: Psychological status analysis, establishment and effect evaluation of a comprehensive psychological intervention for patients with cardiac interventional therapy

Random Number：

I volunteered to participate in the study and have signed the informed consent form. I have got a copy of the informed consent form.

**Patient name (printed) Patient signature Date（Y/M/D）**

**Patient contact phone： Patient correspondence address:**

If the patient is unable to sign the ICF because of illiteracy or dysfunction. Can be signed by an independent witness (or family) without the patient himself.

**Independent witness name(printed) Independent witness signature**

**Date（Y/M/D） Independent witness contact phone**

I, the undersigned, have adopted appropriate and accessible language to discussed about the study with participate or legal representative. As far as I am concerned, I have fully informed of the nature and potential benefits and risks in this study, the participate also fully understood the information. I have provided the participate a copy of the informed consent form which have signed the name and date.

**Researcher name (printed) Researcher signature**

**Date（Y/M/D） Researcher contact phone**
