# Supplementary material for: Effects of a psychological intervention programme on mental stress, coping style and immune function in percutaneous coronary intervention patients
Source: PLoS One. 2018 Jan 22;13(1):e0187745. doi: 10.1371/journal.pone.0187745 (PMC5777641; doi:10.1371/journal.pone.0187745)
Supplement: S3 File — (DOC) [file pone.0187745.s003.doc]

哈尔滨医科大学生物医学研究

伦理审查报告

审查编号：HMUIRB20120009

哈尔滨医科大学附属第二医院心内科拟开展“心脏介入诊疗术患者的心理状态分析及综合心理干预模式的研究”的科研工作。该项目是以“心脏介入诊疗术患者 ”为研究对象的实验。哈尔滨医科大学医学伦理委员会对该项目的相关医学伦理学问题进行了审查。

**一、项目信息**

研究项目名称：心脏介入诊疗术患者的心理状态分析及综合心理干预模式的研究”

承担单位：哈尔滨医科大学附属第二医院心内科

项目负责人：沈晓颖

职称： 主管护师

研究起止日期： 2009年1月至2014年12月

**二、主要研究内容**

（一）对照组：常规护理，包括入院健康教育、术前准备术后引流管理、切口观察、饮食及活动指导等。

（一）实验组：除接受常规护理外，还接受以下综合心理干预：

1.认知干预

（1）于入院及评估后进行，大约20-30分钟。由受过培训的专业人员（护士）根据患者的年龄、文化程度、病情了解程度等不同情况，有针对性地为患者进行讲解疾病的病因、手术目的及过程、术后注意事项等。

（2）于患者决定手术后进行，大约30-45分钟。组织术前术后患者交流会，鼓励患者积极主动的接受手术与治疗。

（3）于术前一日进行，大于30分钟。患者于床旁观看手术过程及术后注意事项的录像。

2.放松疗法

在住院期间的每日进行放松训练，每次15~30min，每日上午与下午各1次。放松训练方法包括由专业人士进行的渐进式肌肉放松疗法、冥想放松训练、意念引导训练、深呼吸、按摩等。注意，患者每次进行时需要全身肌肉放松，注意力高度集中。

3.情感支持（家庭与社会支持）

渗透在与患者及家属的日常交流中。嘱家属配合护理人员为患者营造一个轻松、充满温情的环境。鼓励患者家属多与患者沟通。此外，邀请行PCI术成功后回访复查住院患者给实验患者传授经验，提供社会支持。

**三、审查评议意见**

经哈尔滨医科大学医学伦理审查委员会审议，该研究的实验设计和实施方案充分考虑了安全性和公平性原则，研究内容不构成对受试者的伤害和风险，受试者的招募计划符合自愿和知情同意原则，并尽最大限度保护受试者隐私，研究内容和结果不存在利益冲突。

**四、结论**

该研究中，受试者权利和利益得到了充分保护，对受试者不存在潜在风险，同意该研究项目按计划进行。

哈尔滨医科大学医学伦理审查委员会

2012年12月31日
