# Supplementary material for: Effects of a psychological intervention programme on mental stress, coping style and immune function in percutaneous coronary intervention patients
Source: PLoS One. 2018 Jan 22;13(1):e0187745. doi: 10.1371/journal.pone.0187745 (PMC5777641; doi:10.1371/journal.pone.0187745)
Supplement: S4 File — (DOC) [file pone.0187745.s004.doc]

**Harbin Medical University**

**Institutional Research Board Report**

**NO.：HMUIRB20120009**

The research on**“Psychological status analysis, establishment and effect evaluation of a comprehensive psychological intervention for patients with cardiac interventional therapy”**intended to carry out in cardiology department, the 2^nd^ affiliated hospital of Harbin Medical University. The program is based on the human sample as the objects of the study. Ethic Board approval from the Human Ethic Review Board, Harbin Medical University is on the process.

**1. Program Information**

**Research project title:** Psychological status analysis, establishment and effect evaluation of a comprehensive psychological intervention for patients with cardiac interventional therapy

**Undertaking project enterprise:** cardiology department, the 2^nd^ affiliated hospital of Harbin Medical University

**Project leader:** Xiaoying Shen; **Position:** supervisor nurses

**Date of the project:** From 1/2009 to 12/2014

1. **Primary content of project**

**control group:** Usual care, the control group received the same usual nursing care, consisting of preoperative preparation, care of drainage tube and incision, and health education of diet and activities, etc..

**experimental group:** In addition to the usual care described above, patients allocated to the intervention group were offered a psychological intervention program.

(1)cognitive therapy:

1. After admission and assessment, explain the cause of disease, purpose and process of operation, postoperative cautions according to age, education and understanding of their own disease and so on (the trained specialist nurse) for 20 to 30 minute.

2)Organize the meeting for postoperative and preoperative patients, and encourage the preoperative patients accept treatment and operation positively and optimistically for 30 to 45 minute, at the day of decision of the operation.

3)plays a videotape about process of operation and postoperative cautions besides the bed for 30 minute before the day of operation.

(2) Relaxation  therapy:

Consists of progressive muscle relaxation, meditation, thoughts guidance, deep breathing, and massage by the specific counseling expert, During the hospitalization period, take the relaxation therapy every day, each 15~30min, 1 times a day in the morning and afternoon.

Note: during the training period, the participants needed to relax the whole body muscle and concentration.

1. emotional support(family and social support)

Created a relaxation and warm environment for patients together with families, and gave them moral encourage and daily care [appropriately](javascript:void(0);). Encouraged patients and families communicate with each other, in the daily communication with patients and family.

In terms of social support, invite post-PCI pass on experience to patients.

1. **Review evaluation opinion**

Safety and fairness principle has been fully considered in the experiment plan. All the volunteers gave written informed consent, and the content of the research have no harm or risk. Before the research was conduct, ethic board approval from Harbin Medical University was obtained. No conflict of interest exist in this study.

1. **Conclusion**

The rights and interests volunteer subjects have been adequately protected in the study, and there is no potential risk to the volunteers. Agree to the study work as planned.

**Institutional Research Board of Harbin Medical University**

31/12/2012
