# Supplementary figures and images for: Effects of a psychological intervention programme on mental stress, coping style and immune function in percutaneous coronary intervention patients
Source: PLoS One. 2018 Jan 22;13(1):e0187745. doi: 10.1371/journal.pone.0187745 (PMC5777641; doi:10.1371/journal.pone.0187745)

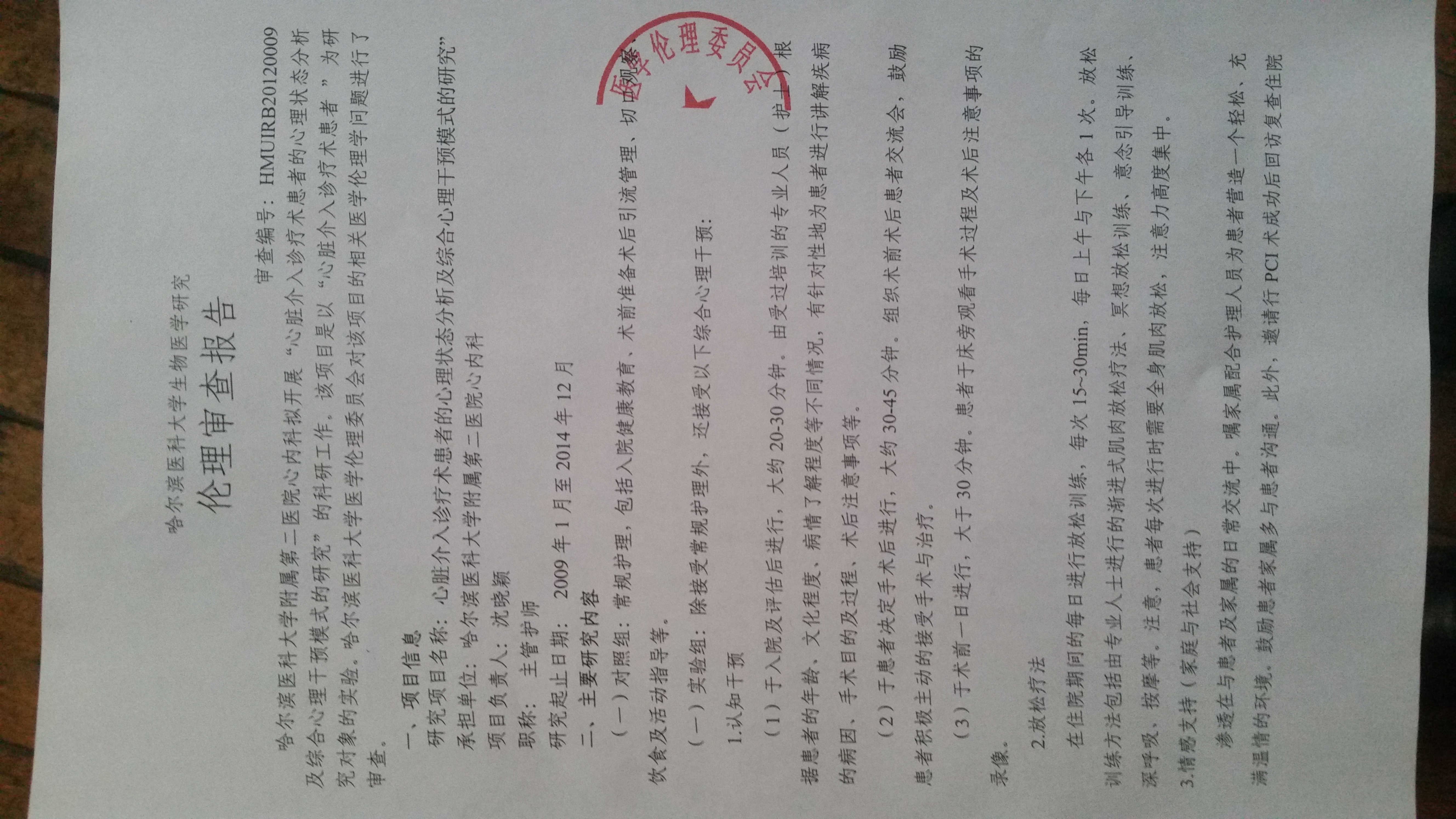

Supplement: S5 File — (JPG) [file pone.0187745.s005.jpg]

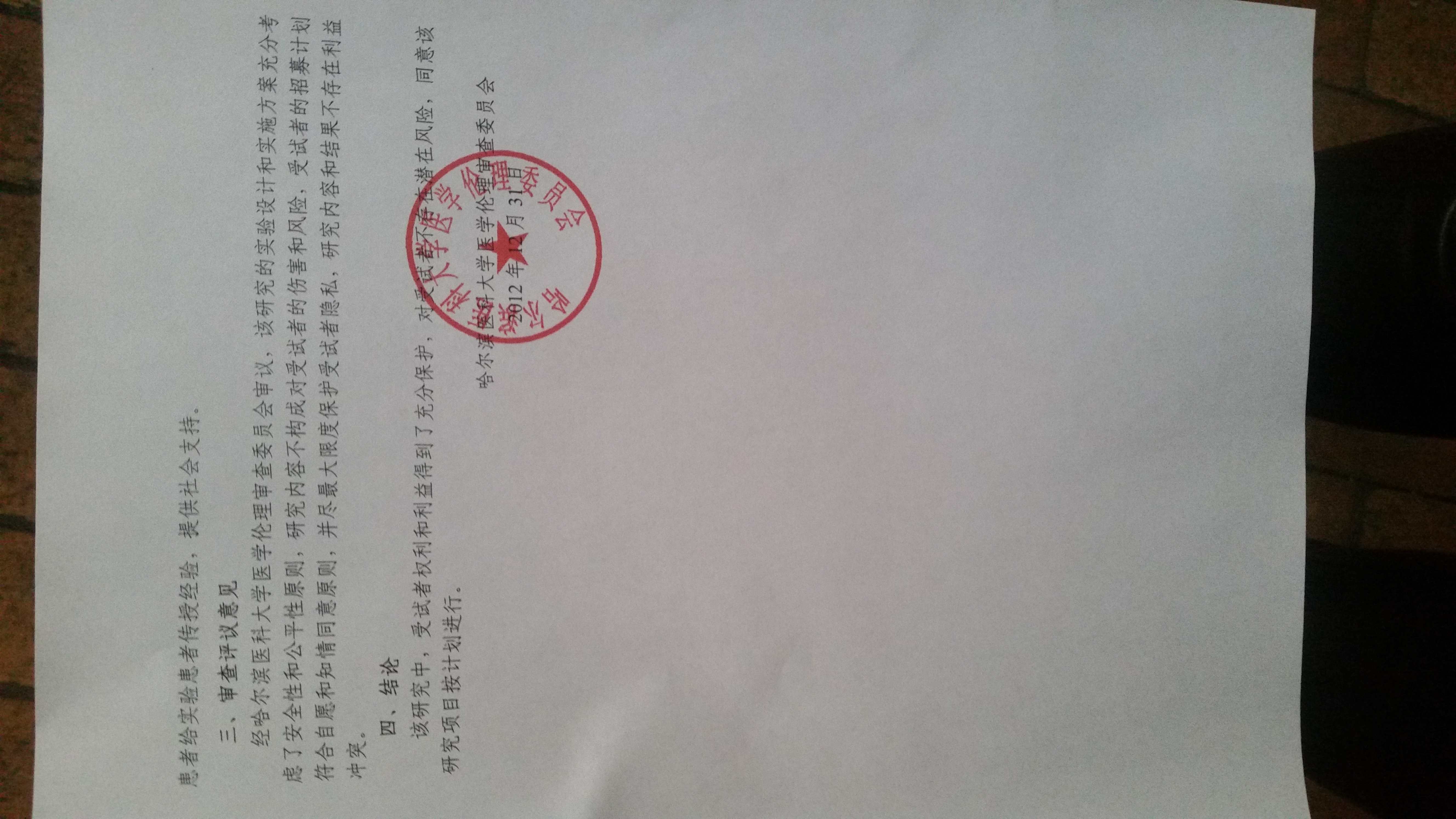

Supplement: S6 File — (JPG) [file pone.0187745.s006.jpg]
