# Supplementary material for: Effects of a psychological intervention programme on mental stress, coping style and immune function in percutaneous coronary intervention patients
Source: PLoS One. 2018 Jan 22;13(1):e0187745. doi: 10.1371/journal.pone.0187745 (PMC5777641; doi:10.1371/journal.pone.0187745)
Supplement: S8 File — (DOCX) [file pone.0187745.s008.docx]

**研究协议**

本研究是一项随机对照临床试验研究设计。

**第一步：研究对象的选取**

拟于2013年8月至2014年5月选取所有在哈医大二院4个病区住院拟行PCI术的冠心病病人，最终纳入60人为止。

1.研究对象的初步入选：在研究对象办理住院时，对患者进行入院评估，从而初步筛选研究对象。具体筛选标准为：①诊断为冠心病，生命体征平稳，择期首次行PCI术。②18-75周岁。③意识清，能用中文进行读写。排除有其他严重循环系统疾病者，有严重并发症者，有其他严重躯体或精神疾病者，有认知障碍者，有原发性或继发性皮质醇增多或减少症者。

2. 进一步筛选研究对象：运用医院焦虑和抑郁量表对满足初筛标准的患者进行问卷调查，筛选出得分大于8分者。

3. 研究者的最终入组：对满足进一步筛选标准的对象介绍研究目的、过程等，邀请其参加研究。自愿参加并签署知情同意书者正式入组。

**第二步：研究对象随机分组**

将最终入组的60例研究对象通过随机数字数列按照1:1的比分入实验组和对照组。随机数字数列由本研究的主持者沈晓颖运用计算机产生。按照入组顺序，研究对象与随机数字数列一一对应，奇数进入实验组，偶数进入对照组。为避免实验组与对照组相互干扰，患者在入组时，安排在不同的病室。所有的研究对象在入组结束后均给予少量的人民币作为报酬。

**第三步：实施干预**

为减少偏倚，本研究采用的是单盲法，实验组和对照组的干预均由课题组成员完成，实验组和对照组的干预人员分别由2个课题组成员组成。对4名成员共同培训，达成一致的标准才进行试验。

将研究对象随机分入实验组（综合心理干预组）和对照组（常规护理组），给予不同的干预，直至患者出院，实验过程为7-14天。

对照组给予常规护理，包括入院健康教育、术前准备术后引流管理、切口观察、饮食及活动指导等。实验组接受常规护理外，还接受以下综合心理干预：

1.认知干预

（1）于入院及评估后进行，大约20-30分钟。由受过培训的专业人员根据患者的年龄、文化程度、病情了解程度等不同情况，有针对性地为患者进行讲解疾病的病因、手术目的及过程、术后注意事项等。

（2）于患者决定手术后进行，大约30-45分钟。组织术前术后患者交流会，鼓励患者积极主动的接受手术与治疗。

（3）于术前一日进行，大于30分钟。患者于床旁观看手术过程及术后注意事项的录像。

2.放松疗法

在住院期间的每日进行放松训练，每次15~30min，每日上午与下午各1次。放松训练方法包括由专业人士进行的渐进式肌肉放松疗法、冥想放松训练、意念引导训练、深呼吸、按摩等。注意，患者每次进行时需要全身肌肉放松，注意力高度集中。

3.情感支持（家庭与社会支持）

渗透在与患者及家属的日常交流中。嘱家属配合护理人员为患者营造一个轻松、充满温情的环境。鼓励患者家属多与患者沟通。此外，邀请行PCI术成功后回访复查住院患者给实验患者传授经验，提供社会支持。

**第四步：指标测量**

研究中所有测量数据的收集与分析均由另一位不了解分组情况的课题组成员进行。所有数据均录入两次，核对无误才可进行分析。

1.人口统计学资料及临床资料的收集。在入院当日，采用问卷调查的方式收集人口学资料和病史资料。所有的问题都是封闭式问题，包括4个人口统计学问题（年龄，性别，婚姻，教育）和1个医学史的问题（是否有血管内支架）。另外还从患者的病历中记录研究对象的5个临床化验指标（血红蛋白、总胆固醇、甘油三酯、高密度脂蛋白、低密度脂蛋白）。

2.心理指标的自我评估。采用1对1现场问卷调查的方式进行SCL-90和MCMQ的调查。在整个研究过程中进行两次此自我评估的问卷调查：在干预开始前（前测），即，入院当天，和计划完成后（后测），即出院前一天。

（1）症状自评量表SCL-90。1975由L.R. Derogatis建立，该量表共有90个项目，分为10个因子分别反映有无各种心理及其严重。这10各因子分别是躯体化、强迫、人际关系敏感、抑郁、焦虑、敌对、偏执、精神病性、恐怖等。项目评分标准：采用五级评分法（0～4级）0分=无：自觉本周内无此问题或症状；1分=轻度：自觉有此问题或症状，但发生得不频繁，程度较轻，持续时间较短：2分=中度：自觉常有此问题或症状，频度和严重度均大于“轻度”。3分=偏重：自觉多数时间均有此问题或症状， 其频度和严重性对自己的学习或生活已产生一定的影响。4分=严重：自觉绝大部分时间都有此问题或症状，自感频度和强度已十分严重。

（2）应对方式MCMQ。医学应对问卷由Feifel H研制，沈晓红等人修订。对包含20个项目，覆盖的三个维度，即面对、回避、屈服，符合人们面临危险事件的基本反应方式。条目评分采用Likert 4级评分，从1分“从不这样”到4分“总是这样”，其中有8条为反向记分，各维度由相应条目分累计所得，得分越高代表病人采用该分量表代表的应对方式的倾向性就越大。

3.生理指标的测量。采集血液标本，测量IL-2和皮质醇。分别在入院第二日和出院当日的早6点进行标本的采集，要求患者至少空腹6-8小时。血液样本被送往内分泌实验室进行分析。实验材料及血液标本的采集：碘[^125^I]皮质醇（Cor）放射免疫试剂盒由北京科美东雅生物技术有限公司提供。碘[^125^I]白细胞介素放射免疫试剂盒-2由中国人民解放军301医院科技考法中心提供。
