# Supplementary material for: Effects of a psychological intervention programme on mental stress, coping style and immune function in percutaneous coronary intervention patients
Source: PLoS One. 2018 Jan 22;13(1):e0187745. doi: 10.1371/journal.pone.0187745 (PMC5777641; doi:10.1371/journal.pone.0187745)
Supplement: S9 File — (DOCX) [file pone.0187745.s009.docx]

Research protocol

This study is a randomized controlled clinical trial design.

**Step one: Select research object.**

From August 2013 to August 2014, patients with coronary heart disease who admitted to 4 wards of No.2 Affiliated Hospital of Harbin Medical University will be recruited, and at last 60 will be enrolled.

*1. Preliminary selection of research objects.*

When the subjects were hospitalized, patients were assessed for admission, and a preliminary screening object will be conducted. The inclusion criteria included individuals who 1) were diagnosed with CAD, had stable vital signs and were scheduled for PCI for the first time, 2) were aged from 18-75 years, 3) were conscious and able to read and speak in Chinese. Patients who suffered from other serious circulatory system diseases or had serious complications, had other serious somatic or mental illness, were cognitively impaired, or had primary or secondary hypercortisolism and cortisol thrombocytopenia were excluded.

*2. Further screening of research objects.*

The hospital anxiety and depression scale will be used to investigate those met the screening criteria, the score greater than 8 points will be screened out.

*3. Final screening of research objects.*

All objects meet the screening criteria above will be invited to participate the study, after being introduced the research purpose, process etc.. Those voluntarily participated in and signed the informed consent form will become formal objects of study.

**Step two: Random grouping of research objects.**

Participants will be randomly assigned in a 1:1 ratio to either an experimental or a control group by random sequence number generation. The random number sequence will be generated by the project leader, Xiaoying Shen, using a computer. All subjects will be assigned into a different group by a random number; those with an odd number will entere the experimental group, and even into the control group. In order to avoid the interference between the experimental group and the control group, the patients in different group will be arranged in different room. All participants will be given a small gift (RMB) after participation.

**Step three: Implementing interventions.**

In order to reduce the bias, this study will use a single blind method. Intervention of experimental group and control group will be completed by member of research team. The intervention implementers of experimental group and control group are the different two members of research team. The four members will be trained until they met the consistent standard.

The subjects will be randomly divided into experimental group (comprehensive psychological intervention group) and control group (conventional care group), and will be given different intervention until the patient discharged from the hospital, the experimental process will be 7-14 days.
Both the control and the experimental group will receive the same usual nursing care, consisting of preoperative preparation, care of drainage tube and incision site, and health education concerning diet and activities. In addition to the usual care described above, patients allocated to the intervention group will be offered a psychological intervention programme.

*1. Cognitive therapy.*

(1) After admission and assessment, explain the cause of disease, purpose and process of operation, postoperative cautions according to age, education and understanding of their own disease and so on (the trained specialist nurse) for 20 to 30 minute.

(2) Organize the meeting for postoperative and preoperative patients, and encourage the preoperative patients accept treatment and operation positively and optimistically for 30 to 45 minute, at the day of decision of the operation.

(3) Plays a videotape about process of operation and postoperative cautions besides the bed for 30 minute before the day of operation.

*2. Relaxation therapy.*

Consists of progressive muscle relaxation, meditation, thoughts guidance, deep breathing, and massage by the specific counseling expert, During the hospitalization period, take the relaxation therapy every day, each 15~30min, 1 times a day in the morning and afternoon.

Note: during the training period, the participants needed to relax the whole body muscle and concentration.

*3. Emotional support (family and social support).*

Created a relaxation and warm environment for patients together with families, and gave them moral encourage and daily care [appropriately](javascript:void(0);). Encouraged patients and families communicate with each other, in the daily communication with patients and family.

In terms of social support, invite post-PCI pass on experience to patients.

**Step four: Measurement index.**

The collection and analysis of all the data will be carried out by another team member who does not understand the grouping arrangement.

*1. Demographic data and clinical data.*

On the day of admission, we will adopt self-made questionnaire to investigate and collect the demographic data and medical history data. All questions are close-ended, including 4 demographic questions (age, sex, marriage, education), and 1 medical history questions (if there is a vascular stent). In addition, 5 clinical laboratory indexes (hemoglobin, total cholesterol, triglyceride, high density lipoprotein, low density lipoprotein cholesterol, low density lipoprotein cholesterol, low density lipoprotein) were recorded from the patient's medical records.

*2. Psychological indicator.*

A 1 to 1 spot field survey will be conducted to investigate SCL-90 and MCMQ. Two such questionnaires will be conducted throughout the study: that is prior to the start of the intervention (pre-test), i.e., on the day of admission, and after completion of the intervention (post-test), i.e., the day before discharge.

(1) Self-Report Symptom Checklist (SCL-90): The SCL-90 was established by L.R. Derogatis in 1975. It is composed of 90 self-reported items, 9 factors on symptoms and 1 factor that assesses additional items. This scale contains of various items on feeling, thinking, awareness, behaviour, life habits, interpersonal relationships, diet, sleep, etc. The ten factors are somatization, obsessive-compulsive, interpersonal sensitivity, depression, anxiety, hostility, paranoid ideation, psychoticism, phobic anxiety, and other. The scale adopts Likert 5-point, from 0 point to 4 point. 0 points means never: no symptoms or problem within this week. 1 point means mild: consciously have this kind of problem or symptoms, but it is of not frequent incidence, of light degree and short duration. 2 point means moderate: consciously often have this kind of problem or symptoms, the frequency and severity are greater than the mild. 3 means more serious, consciously majority of time have this kind of problem or symptoms, the frequency and severity have a certain impact on your study or life. 4 means severe: consciously most of times have such kind of problems or symptoms, the frequency and severity is very serious.

(2) Medical Coping Mode Questionnaire (MCMQ): The MCMQ was established by Feifel H, revised by Shen Xiaohong et al. It contains 20 items covering three dimensions, namely confrontation, avoidance, and suppression, which reflect the basic reactions of people at risk. The scale adopts Likert 4-point, from 1 point (Never like this) to 4 point (always like this). It has 8 reverse scoring items. The scores of each dimension are accumulated by score of each item, the higher the score, the greater the tendency of the patient to adopt the coping style the sub-scale presented.

*3. Physiological indicators.*

IL-2 and cortisol were measured in the blood at two time points as mentioned. To avoid physiological for analysisy mission)ping stying stype. ts needed to relax the whole body muscle and concentration. fluctuations, the IL-2 and cortisol specimens were collected at 6 AM, when the patients had an empty stomach. Blood samples were sent to the endocrine laboratory for analysis. For cortisol, an iodine (125I) glycol (Cor) RIA kit was provided by the Beijing Kemeidongya biological technology co., LTD.
